# Deregulation of miR‐1245b‐5p and miR‐92a‐3p and their potential target gene, *GATA3*, in epithelial–mesenchymal transition pathway in breast cancer

**DOI:** 10.1002/cnr2.1955

**Published:** 2024-01-03

**Authors:** Mahtab Yadollahi Farsani, Zeinab Amini Farsani, Shohreh Teimuri, Mohsen Kolahdouzan, Reza Eshraghi Samani, Hossein Teimori

**Affiliations:** ^1^ Department of Medical Biotechnology, School of Advanced Technologies Shahrekord University of Medical Sciences Shahrekord Iran; ^2^ Cellular and Molecular Research Center, Basic Health Sciences Institute Shahrekord University of Medical Sciences Shahrekord Iran; ^3^ Institute of Cell Biology University of Bern Bern Switzerland; ^4^ Department of Surgery, School of Medicine Isfahan University of Medical Sciences Isfahan Iran

**Keywords:** breast cancer, epithelial‐mesenchymal transition, *GATA3*, MicroRNAs

## Abstract

**Background:**

MicroRNAs (miRNAs) are small molecules that have prominent roles in tumor development and metastasis and can be used for diagnostic and therapeutic purposes. This study evaluated the expression of miR‐92a‐3p and miR‐1245b‐5p and their potential target gene, *GATA3* in patients with breast cancer (BC).

**Materials and Methods:**

In the search for BC‐related microRNAs, miR‐124b‐5p and miR‐92a‐3p were selected using Medline through PubMed, miR2disease, miRcancer and miRTarBase. Moreover, target gene *GATA3* and their possible interaction in the regulating epithelial‐mesenchymal transition (EMT) and invasion was evaluated using in silico tools including miRTarBase, TargetScan, STRING‐db, and Cytoscape. The expression level of miR‐92a‐3p, miR1245b‐5p, and *GATA3* were assessed on extracted RNAs of tumor and nontumor tissues from 36 patients with BC using qPCR. Additionally, clinical‐pathologic characteristics, such as tumor grade, tumor stage, lymph node were taken into consideration and the diagnostic power of these miRNAs and *GATA3* was evaluated using the ROC curve analysis.

**Results:**

In silico evaluation of miR‐92a‐3p and miR‐1245b‐5p supports their potential association with EMT and invasion signaling pathways in BC pathogenesis. Comparing tumor tissues to nontumor tissues, we found a significant downregulation of miR‐1245b‐5p and miR‐92a‐3p and upregulation of *GATA3*. Patients with BC who had decreased miR‐92a‐3p expression also had higher rates of advanced stage/grade and ER expression, whereas decreased miR‐1245b‐5p expression was only linked to ER expression and was not associated with lymph node metastasis. The AUC of miR‐1245b‐5p, miR‐92a‐3p, and *GATA3* using ROC curve was determined 0.6449 (*p* = .0239), 0.5980 (*p* = .1526), and 0.7415 (*p* < .0001), respectively, which showed a significant diagnostic accuracy of miR‐1245b‐5p and *GATA3* between the BC patients and healthy individuals.

**Conclusion:**

MiR‐1245b‐5p, miR‐92a‐3p, and *GATA3* gene contribute to BC pathogenesis and they may be having potential regulatory roles in signaling pathways involved in invasion and EMT pathways in BC pathogenesis, as a result of these findings. More research is needed to determine the regulatory mechanisms that they control.

## 1 INTRODUCTION

Breast cancer (BC), as a multifactorial disease, is the leading cause of death in women, globally.^1^ Despite the numerous studies on BC, the molecular mechanisms of this disease have not been fully known.^2^, ^3^ The survival rate of the patient in the early stages of this cancer is high, therefore early diagnosis of BC is very crucial.^4^, ^5^ Moreover, suggesting the potential biomarkers for early diagnosis is a promising strategy.^5^, ^6^

MicroRNAs (miRNAs), as highly conserved single‐strand short RNAs, link with 3'UTR regions in their target genes to regulate gene expression in the transcription level.^7^ Recent studies showed that the specific miRNAs act as potential oncogenes or tumor suppressors in human breast tumors. Accordingly, the targeting miRNAs or their associated targets can modulate various biological activities of cancer cell, such as cell metabolism, proliferation, and invasion.^8^, ^9^, ^10^, ^11^ The cancer invasion and metastasis program aim to investigate the underlying processes that are responsible for the majority of cancer deaths.^12^, ^13^ Epithelial–mesenchymal transition (EMT) is a leading cause of distant metastases in the epithelial cancers, such as BC. EMT able to disrupt cell‐to‐cell junctions, thereby leading to the gain of mesenchymal shape and loss of epithelial properties. As a result, cells are released from the parental epithelial tissues and regenerate new metastatic tissues. miRNAs can target EMT‐inducing transcription factors such as basic helix loop helix (bHLH), SNAIL, TWIST, and ZEB and inhibit the EMT process.^14^ For example, miR‐200 targets ZEB1 and ZEB2 mRNAs in a direct manner via the upregulation of E‐cadherin in cancer cell lines and thus suppresses cell motility and EMT pathway.^15^ Therefore, the prediction and identification of miRNAs contributing to EMT, can be introduced as diagnostic and therapeutic tools in the treatment of various cancers.

Recently, it has been reported that the weak prognosis in cancer patients is associated with the upregulation of miR‐92a in blood or tumor tissues miR‐92a. As, several studies reported that patients with high expression levels of miR‐92a experience lower survival rates or faster tumor progression and metastasis in colorectal cancer^16^, ^17^, ^18^ and nonsmall cell lung cancer.^19^, ^20^ Versus, some new studies showed that the increased expression of miR‐92a is closely related to the patient survival in these cancers. For example, in chronic leukemia, the overexpression of this miRNA was reported to cause higher survival rates in these patients.^21^ The role of this miRNA in BC is also unclear. Some results showed that an increased expression of this miRNA is associated with decreased tumor macrophage infiltration and better outcomes in BC.^22^ On the contrary, another study indicated that the expression of miR‐92a is directly related to the tumor size and increased TNM stage of BC.^23^ Moreover, there are limited studies on the *in‐silico* prediction of the role of miR‐92a‐3p in the regulating EMT and invasion in the BC.

The miR‐1245b family consists of the miR‐1245a and miR‐1245b, both of which are expressed in breast tissue. Limited research has been conducted on their roles in various cancers. Recently, Yan et al showed that the inhibition of miR1245b‐5p in osteosarcoma cancer significantly increased the invasiveness in this cancer.^24^ Moreover, MiR‐1245 upregulation in BC represses homologous recombination (HR)‐mediated repair and increases the cancer cell sensitivity to γ‐irradiation by the targeting the tumor suppressor gene, BRCA2.^25^ There is no information on miR‐1245b and its potential role in the regulating the EMT pathway and BC invasion. This study evaluated the expression levels of miR‐29a‐3p, miR‐1245b‐5p, and *GATA3* in the BC and normal tissues and investigate their association with clinicopathological characteristics of the tumors. We also evaluated the diagnostic power of *GATA3* with miR‐92a‐3p and miR‐1245b‐5p through the ROC curve. In addition, using in silico tools, we addressed the possible role of these miRNAs in regulating other genes implicated in EMT and invasion in BC pathogenesis.

## 2 MATERIALS AND METHODS

### 2.1 In silico analysis

Along with a comprehensive literature review in an electronic database, MEDLINE, through PubMed (the U.S. National Library of Medicine and the National Institutes of Health), genes contributing in the EMT and invasion signaling pathways in BC cells were searched using KEGG (https://www.genome.jp/kegg) and WikiPathways (https://www.wikipathways.org) databases. The expression of searched genes was investigated in the UniGene and the human protein atlas (https://www.proteinatlas.org/databases), and genes with specific high expression in the breast tissue were selected. Next, the probable target miRNAs for the selected genes were assessed using PicTar (https://pictar.mdc-berlin.de/cgibin/PicTar_vertebrate.cgi), miRTarBase (http://mirtarbase.mbc.nctu.edu.tw/php/index.php),^26^ miRWalk (mirwalk.umm.uni‐heidelberg.de) and TargetScan Human Version 7.2 (www.targetscan.org),^27^ and prepared a list of miRNAs that their role in the regulating the EMT phenomenon in BC had not been extensively investigated.^24^, ^28^, ^29^, ^30^, ^31^ Furthermore, the expression of selected miRNAs and also their link with BC were checked in different literature and miRTarBase database, and using miR2disease, miRcancer, and breast‐originated diseases (miRTarBase), respectively.

The protein–protein interactions of the potential target genes with selected miRNAs were investigated by STRING‐db (https://string-db.org), and results were visualized in Cytoscape V.1.7.3 software. The number of direct edges was used in Cytoscape to visualize and analyze the network for constructing the network graph. Furthermore, a bioinformatics analysis was performed to determine the possible function of the desired miRNAs in the regulating other genes involved in general signaling pathways related to invasion and EMT. An enrichment assessment was performed for the target genes of desired miRNAs by DIANA miRPath v.3 tools.^32^

### 2.2 Preparation of normal and cancerous breast tissues

Both normal and cancerous breast tissue samples were collected from 36 women aged 27–85 undergoing surgery between October 2018 and April 2019 at Seyed‐O‐Shohada Hospital in Isfahan and Ayatollah Kashani hospital in Shahrekord, both in Iran. All patients had not taken any adjuvant therapy before surgery. The subjects under treatment with chemotherapy and radiotherapy were excluded of study. The written informed consent was signed by volunteers and frozen tissue specimens were stored at −80°C for further research. A professional pathologist characterized all the patients. The clinicopathological characteristics of these patients are detailed in Table 1. This study was approved by the Institutional Review Board (IRB) of the Shahrekord Universities of Medical Sciences.

**TABLE 1:** The clinicopatological characteristics of BC patients and tissues.

Variables	Frequency (%)
Age	
≤ 50 years	21 (58.33%)
> 50 years	15 (41.66%)
Tumor size (cm)	
≤2	8 (22.22%)
2–5	19 (52.77%)
>5	9 (25%)
Tumor stage	
Stage I/ II	27 (75%)
Stage III	9 (25%)
Histological grade	
Grad I/II	24 (66.66%)
Grad III	12 (33.33%)
Lymph node status	
Absent	18 (50%)
Present	18 (50%)
ER status	
Positive	29 (80.55%)
Negative	7 (19.44%)
Subtypes	
Luminal A	17 (47.22%)
Luminal B	12 (333.33%)
HER2	3 (8.33%)
Triple negative	4 (11.11%)

### 2.3 Total RNA isolation

The breast tissue samples were subjected to RNX‐Plus solution (1 mg/50 mg breast tissue) based on the manufacturer's protocol (SinaClon BioSience, Iran). Tissue lysis was gently carried out with a homogenizer for 30 min at 37°C. Subsequently, RNA quality and quantity were evaluated using Nano drop and agarose gel. Only those samples with two distinct rRNA bands (28s, 18s) and 260/280 ratio of 1.8–2 were considered for further analysis. The specimens were kept at −80°C until further investigation.

### 2.4 cDNA synthesis

Treated RNA with DNase I, was used for cDNA synthesis using a cDNA synthesis kit (Yektatajhis, Tehran, Iran cat No. Y. TA500). The reaction in a final volume of 10 μL containing 500 ng RNA, 0.5 μL RT enzyme, 2 μL 5 × prime script buffer, 0.5 μL random 6mer, and 0.5 μL oligo dT primer was incubated for 60 min at 42°C, 60 min at 37°C, and 5 min at 70°C. Moreover, cDNA synthesis for miRNA was performed using a cDNA synthesis kit (Bonyakhteh, Tehran, Iran, cat. no: Bon 209001) through polyadenylation of the 3′ end of all the RNAs. Briefly, elongation was performed in a polyadenylation reaction, with a final volume of 20 μL at 37°C for 30 and 20 min at 65°C. Then the reaction to make cDNA was performed immediately after polyadenylation reaction and using the existing compounds in cDNA Synthesis Kit, for each sample of polyadenylated RNA. cDNA specimens were kept at −80°C.

### 2.5 Real‐time quantitative polymerase chain reaction for miRNA and target gene

A set of primers were designed by Primer 3 software (F: 5'ATGCCTGCCGTGTGAAC3' and R: 5'ATCTTCAAACCTCCATGATG3' for *β2M*) and (F: 5'GAGACAGAGCGAGCAACG3' and R: 5'CTCGGGTCACCTGGGTAG3' for *GATA3*) to amplify the *β2M* and *GATA3* gene. The quantitative real‐time RT‐PCR was carried out using YTA SYBER Green qPCR Master Mix 2 × (Cat# YT2551, Yekta Tajhiz Azma, Tehran, Iran) and BONmiR High‐Specificity miRNA qPCR Core Reagent Kit (Cat# 209002, Bonyakhteh, Tehran, Iran) to detect the mRNA and miRNA levels. The thermal program for carrying out *GATA3* gene amplification was set as 95°C for 3 min, and then 40 cycles for 15 s at 95°C, for 30s at 60°C, and for 20s at 72°C. Amplification of miR‐92a was carried out for 2 min at 95°C, 40 cycles for 15 s at 95°C, and for 30s at 60°C. Results were analyzed according to the Livak or 2^−ΔΔCt^ method, followed by normalization for *β2M* and C/D box snoRNAs (SNORD) as an internal control for genes and miRNAs, respectively.

### 2.6 Statistical analyses

Data analysis was performed with GraphPad Prism V.6 software (GraphPad; USA). Data were reported as mean ± standard deviation. One‐way analysis of variance (ANOVA) followed by Dunnett's multiple comparison test was carried out by analyzing several groups, and an independent Student's *t*‐test was used to compare cancerous vs. control tissues. Pearson's correlation analysis was also performed for the miRNA‐mRNA co‐expression. Moreover, receiver operating characteristic (ROC) curve analysis was conducted to investigate the diagnostic power of *GATA3* and miRNAs. *p*‐values <.05 were considered statistically significant.

## 3 RESULTS

### 3.1 In silico analysis

**FIGURE 1:**
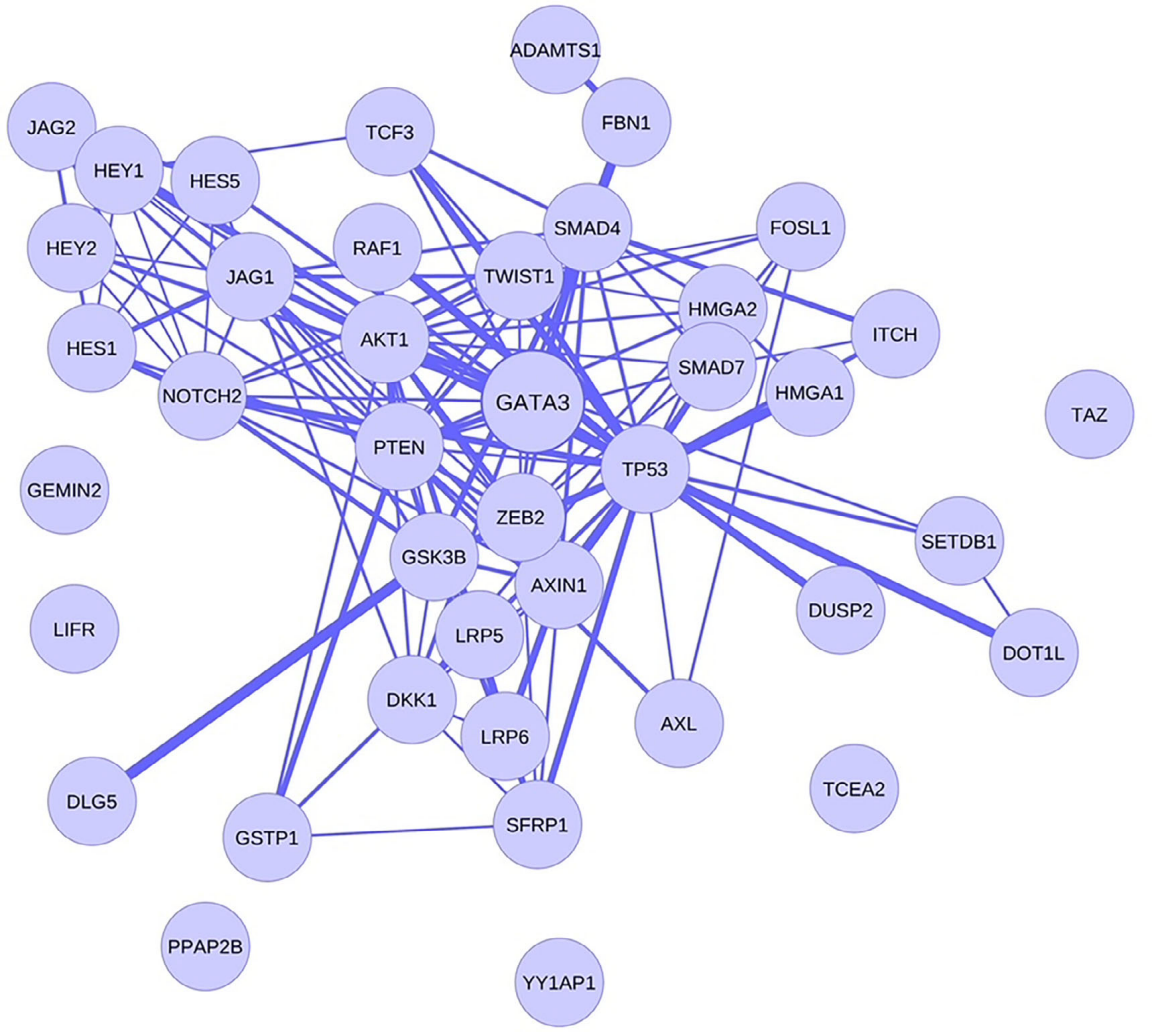
Interactions of genes implicated in BC pathogenesis and predicted targets of miR‐92a‐3p and miR‐1245b‐5p using STRING db. As the line thickness increases, the interaction of proteins with each other also increases. It is predicted that GATA3 interacts with many proteins participating in the EMT and invasion pathways involved in BC pathogenesis.

**FIGURE 2:**
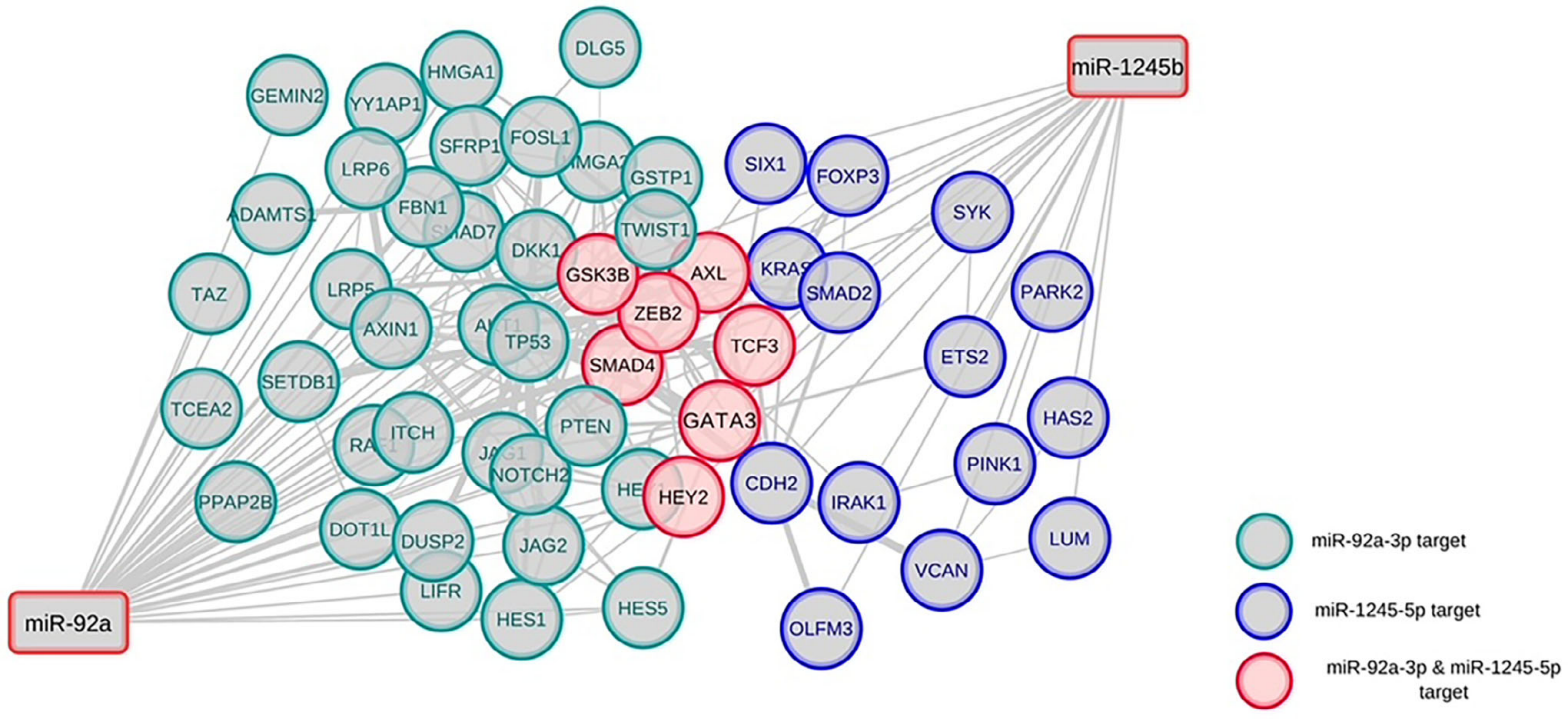
BC‐related genes that may interact with miR‐1245b‐5p and miR‐92a‐3p were visualized in Cytoscape after inputting to STRING‐db. Genes in green, blue, or red circles display the targeted genes of miR‐92a‐3p, miR‐1245b‐5p, or both of them, respectively.

**FIGURE 3:**
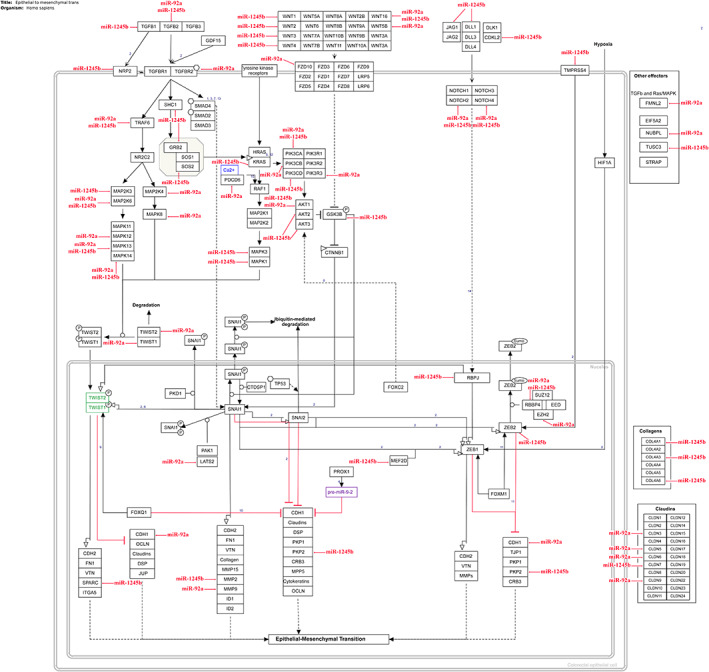
Potential targets of miR‐92a‐3p and miR‐1245b‐5p during the EMT process.

**FIGURE 4:**
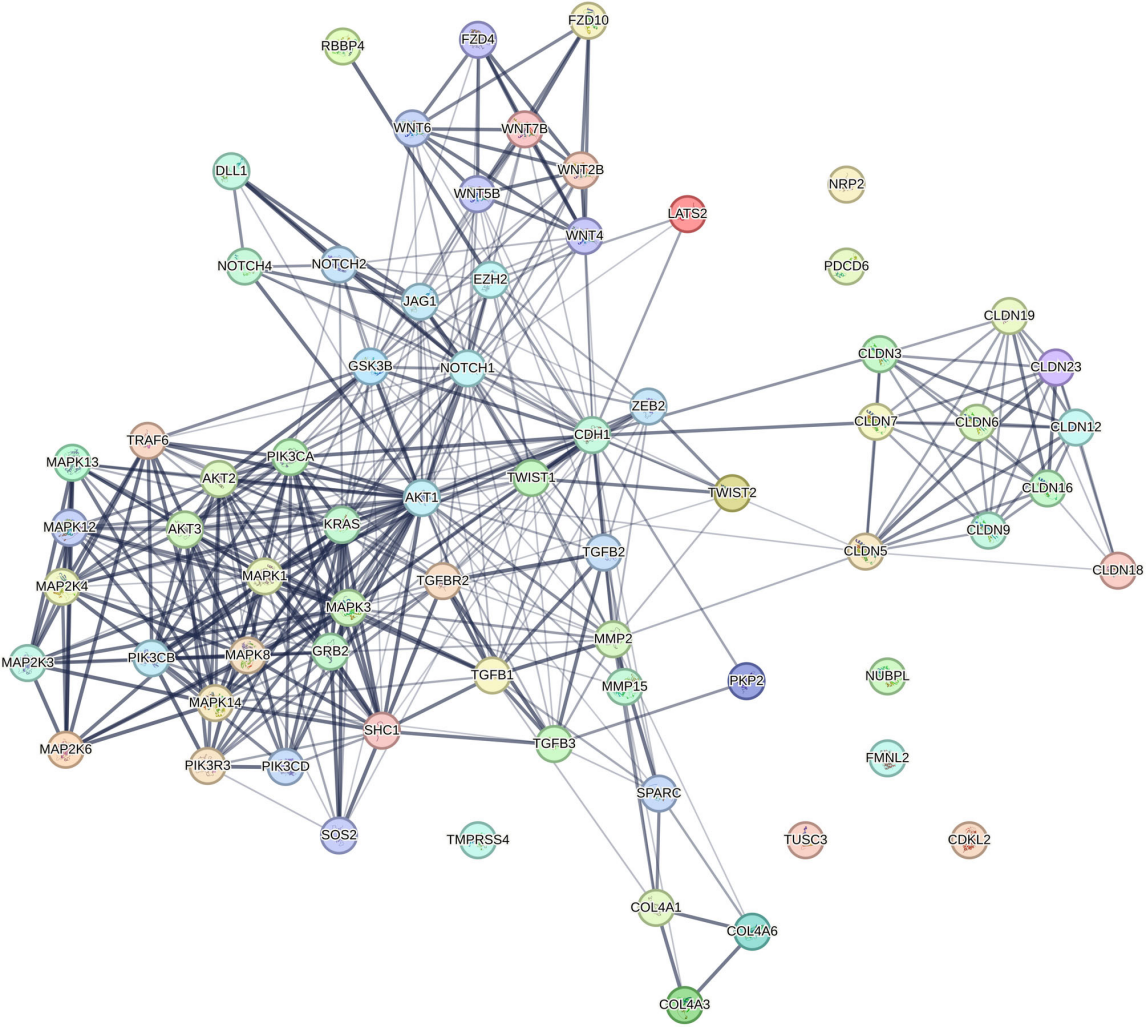
The potential interactions of genes implicated in the EMT process using the STRING db. The thickness of the lines indicates the interaction level of genes with each other.

**FIGURE 5:**
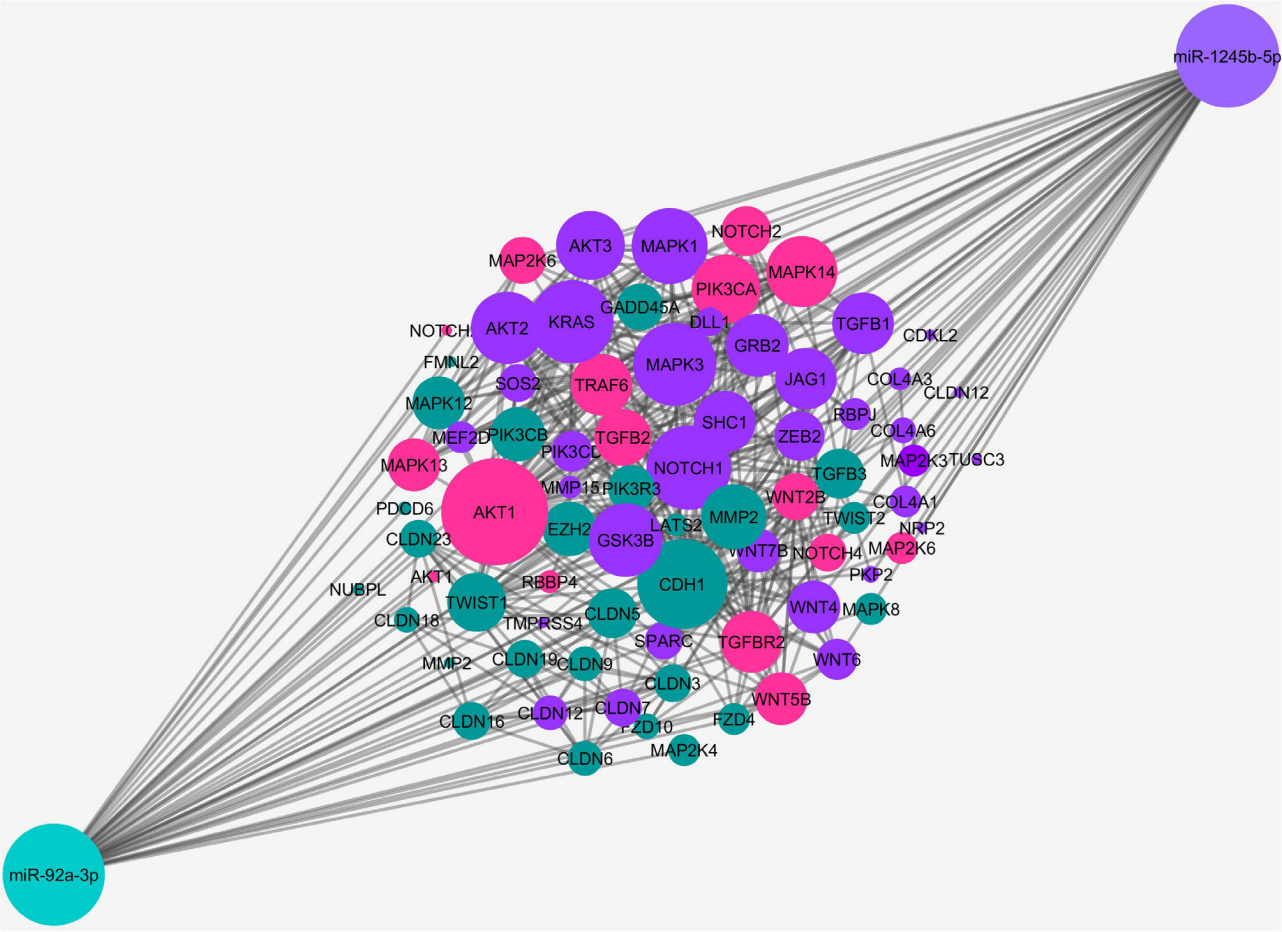
The potential interactions of genes implicated in the EMT process with miR‐92a‐3p and miR‐1245b‐5p. (in databases including WikiPathways, MiRWalk, MirBase, Target scan, and MiRTarBase). The potential target genes of miR‐92a‐3p (green), miR‐1245b‐5p (purple), and common targets (pink) are shown in the picture.

**FIGURE 6:**
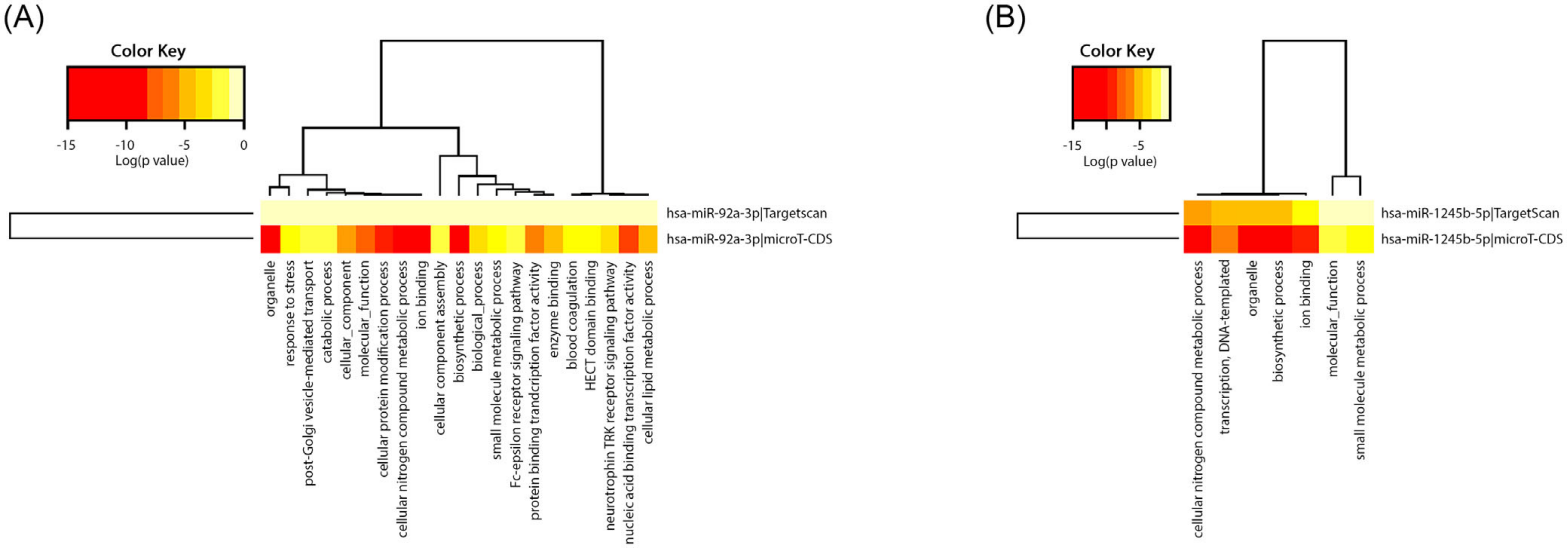
The functions of miR‐92a‐3p and miR‐1245b‐5p in cancer progression were elucidated by gene ontology enrichments of their target genes via miRPath v3.0 based on TargetScane and microT‐CDS. The color intensity (red to yellow) indicates the extent to which the genes of this pathway are regulated by the mentioned miRNAs. As the color intensity increases, the effect of that miRNA on the signaling pathway increases.

The KEGG and WikiPathways databases were searched for finding genes contributing to the EMT and invasion signaling pathways in BC cells. Moreover, the expression of searched genes was evaluated in the UniGene and the human protein atlas and the *GATA3* gene was chosen for further in vitro investigation. *GATA3* gene with ability to regulate cancer‐related signaling pathways out of all the existing genes, is the sixth most important mutated gene in BC in the TCGA (The Cancer Genome Atlas Program) database (https://portal.gdc.cancer.gov) which has a high specific expression in breast tissue and limited studies have been done on it. Furthermore, using PicTar, miRTarBase, miRWalk, and TargetScan Human Version 7.2 to find the probable target miRNAs for the *GATA3* gene, miR‐92a‐3p and miR‐1245b‐5p were selected considering high binding score to *GATA3* gene, their predicted significant relationship with the breast‐related diseases and limited study in the EMT phenomenon in BC. Other important genes in the BC invasion which are potentially regulated by desired miRNAs are detailed in Table 2. Moreover, expressed genes in the breast tissue that contribute to EMT and invasion and are potential targets of the desired miRNAs were summarized in Table 3, and were subjected to STRING‐db v11 and visualized by Cytoscape (Figures 1, 2, 3, 4, 5). The pathways associated with desired miRNAs were determined by miRPath v3.0 and the output was depicted in the heatmap (Figure 6).

**TABLE 2:** Potential targets of miR‐92a and miR‐145b involved in EMT pathway.

Gene	UniGene ID	miRNA	Full name of the genes
TWIST1	Hs.7291	miR‐92a	Twist family bHLH transcription factor 1
ZEB2	Hs.9839	miR‐92a,miR‐1245b	Zinc finger E‐box binding homeobox 2
GEMIN2	Hs.8487	miR‐92a	Gem nuclear organelle associated protein 2
AXL	Hs.558	miR‐92a	AXL receptor tyrosine kinase
PTEN	Hs.5728	miR‐92a	phosphatase and tensin homolog
Smad7	Hs.4092	miR‐92a	SMAD family member 7
FAR‐1	Hs.84188	miR‐92a	Fatty acyl‐CoA reductase 1
SETDB1	Hs.9869	miR‐92a	SET domain bifurcated histone lysine methyltransferase 1
GATA3	Hs.2625	miR‐92a,miR‐1245b	GATA binding protein 3
ADAMTS1	Hs.9510	miR‐92a	ADAM metallopeptidase with thrombospondin type 1 motif 1
HMGA2	Hs.8091	miR‐92a	High mobility group AT‐hook 2
GSK3B	Hs.2932	miR‐92a	Glycogen synthase kinase 3 beta
Smad4	Hs.4089	miR‐92a	SMAD family member 4
TP53	Hs.7157	miR‐92a	Tumor protein p53
EPHA8	Hs.2046	miR‐92a	EPH receptor A8
PLPP3	Hs.8613	miR‐92a	Phospholipid phosphatase 3
FBN1	Hs.2200	miR‐92a	Fibrillin 1
PLAT	Hs. 5327	miR‐92a	plasminogen activator, tissue type
RAB8B	Hs.235442	miR‐92a	RAB8B, member RAS oncogene family
MAPK3	Hs.5595	miR‐92a	Mitogen‐activated protein kinase 3
MAPK1	Hs.5594	miR‐92a	Mitogen‐activated protein kinase 1
TCF3	Hs.6929	miR‐92a	Transcription factor 3
HEY1	Hs.23462	miR‐92a	Hes related family bHLH transcription factor with YRPW motif 1
HES1	Hs.3280	miR‐92a	Hes family bHLH transcription factor 1
YAP1	Hs.10413	miR‐92a	Yes1 associated transcriptional regulator
TAZ	Hs.6901	miR‐92a	Tafazzin
STK4	Hs.6789	miR‐92a	Serine/threonine kinase 4
HMGA1	Hs.3159	miR‐92a	High mobility group AT‐hook 1
ACT1	Hs.207	miR‐92a	AKT serine/threonine kinase 1
Raf1	Hs.5894	miR‐92a	Raf‐1 proto‐oncogene, serine/threonine kinase
HEY2	Hs.23493	miR‐92a	Hes related family bHLH transcription factor with YRPW motif 2
Lrp6	Hs.4040	miR‐92a	LDL receptor related protein 6
Axin1	Hs.8312	miR‐92a	Axin1
SFRP1	Hs.6422	miR‐92a	Secreted frizzled related protein 1
DKK1	Hs.22943	miR‐92a	Dickkopf WNT signaling pathway inhibitor 1
JAG1	Hs.182	miR‐92a	Jagged canonical Notch ligand 1
JAG2	Hs.3714	miR‐92a	Jagged canonical Notch ligand 2 1Mitogen‐activated
LIFR	Hs.3977	miR‐92a	LIF receptor subunit alpha Daiblo
ITCH	Hs.83737	miR‐92a	IAP bindingItchy E3 ubiquitin protein ligase
c‐Myc	Hs.4609	miR‐92a	**MYC proto‐oncogene, bHLH transcription factor**
RHOA	Hs.387	miR‐92a	**Ras homolog family member A**
Bmi1	Hs.648	miR‐92a	**BMI1 proto‐oncogene, polycomb ring finger**
GSTP1	Hs.2950	miR‐92a	**Glutathione S‐transferase pi 1**
DOT1L	Hs.84444	miR‐92a	**DOT1 like histone lysine methyltransferase**
SETDB1	Hs.9869	miR‐92a	**SET domain bifurcated histone lysine methyltransferase 1**
OlFM3	Hs.118427	miR‐1245b	**Olfactomedin 3**
SYK	Hs.6850	miR‐1245b	**Spleen associated tyrosine kinase**
TRF6	Hs.7189	miR‐1245b	**TNF receptor associated factor 6**
LUM	Hs.4060	miR‐1245b	**Lumican**
HAS2	Hs.3037	miR‐1245b	**Hyaluronan synthase 2**
KRAS	Hs.3845	miR‐1245b	**KRAS proto‐oncogene, GTPase**
CDH2	Hs.1000	miR‐1245b	**Cadherin 2**
CDKL2	Hs.8999	miR‐1245b	**Cyclin‐dependent kinase like 2**
FOXP3	Hs.50943	miR‐1245b	**Forkhead box P3**
ETS2	Hs.2114	miR‐1245b	**ETS proto‐oncogene 2, transcription factor**
PRKN	Hs.5071	miR‐1245b	**Parkin RBR E3 ubiquitin protein ligase**
VCAN	Hs.1462	miR‐1245b	**versican**
PINK1	Hs.65018	miR‐1245b	**PTEN induced kinase 1**
CXCL16	Hs.58191	miR‐1245b	**C‐X‐C motif chemokine ligand 16**
HEY2	Hs.23493	miR‐1245b	**Hes related family bHLH transcription factor with YRPW motif 2**
SIX1	Hs.6495	miR‐1245b	**SIX homeobox 1**
TCF3	Hs.6929	miR‐1245b	**Transcription factor 3**

**TABLE 3:** Potential targets of miR‐92a‐3p and miR‐1245b‐5p involved in BC invasion.

Gene	miRNA	Full name of the genes
TGFB3	miR‐92a‐3p	Transforming growth factor beta 3
MAPK12	miR‐92a‐3p	Mitogen‐activated protein kinase 12
MAP2K4	miR‐92a‐3p	Mitogen‐activated protein kinase kinase 4
PDCD6	miR‐92a‐3p	Programmed cell death 6
MAPK8	miR‐92a‐3p	Mitogen‐activated protein kinase kinase 8
PIK3CB	miR‐92a‐3p	Phosphatidylinositol‐4,5‐bisphosphate 3‐kinase catalytic subunit beta
PIK3R3	miR‐92a‐3p	Phosphoinositide‐3‐kinase regulatory subunit 3
TWIST2	miR‐92a‐3p	Twist family bHLH transcription factor 2
TWIST1	miR‐92a‐3p	Twist family bHLH transcription factor 1
LATS2	miR‐92a‐3p	Large tumor suppressor kinase 2
CDH1	miR‐92a‐3p	Cadherin 1
MMP2	miR‐92a‐3p	Matrix metallopeptidase 2
EZH2	miR‐92a‐3p	Enhancer of zeste 2 polycomb repressive complex 2 subunit
WNT5B	miR‐92a‐3p	Wnt family member 5B
FZD4	miR‐92a‐3p	Frizzled class receptor 4
FZD10	miR‐92a‐3p	Frizzled class receptor 10
NUBPL	miR‐92a‐3p	Nucleotide binding protein like
FMNL2	miR‐92a‐3p	Formin like 2
CLDN3	miR‐92a‐3p	Claudin 3
CLDN5	miR‐92a‐3p	Claudin 5
CLDN6	miR‐92a‐3p	Claudin 6
CLDN9	miR‐92a‐3p	Claudin 9
CLDN16	miR‐92a‐3p	Claudin 16
CLDN18	miR‐92a‐3p	Claudin 18
CLDN19	miR‐92a‐3p	Claudin 19
CLDN23	miR‐92a‐3p	Claudin 23
TGFB1	miR‐1245b‐5p	Transforming growth factor beta 1
NRP2	miR‐1245b‐5p	Neuropilin 2
SHC1	miR‐1245b‐5p	SHC adaptor protein 1
MAP2K3	miR‐1245b‐5p	Mitogen‐activated protein kinase kinase 3
GRB2	miR‐1245b‐5p	Growth factor receptor bound protein 2
SOS2	miR‐1245b‐5p	SOS Ras/Rho guanine nucleotide exchange factor 2
KRAS	miR‐1245b‐5p	KRAS proto‐oncogene, GTPase
MAPK1	miR‐1245b‐5p	Mitogen‐activated protein kinase 1
MAPK3	miR‐1245b‐5p	Mitogen‐activated protein kinase 3
PIK3CD	miR‐1245b‐5p	Phosphatidylinositol‐4,5‐bisphosphate 3‐kinase catalytic subunit delta
AKT2	miR‐1245b‐5p	AKT serine/threonine kinase 2
AKT3	miR‐1245b‐5p	AKT serine/threonine kinase 3
GSK3B	miR‐1245b‐5p	Glycogen synthase kinase 3 beta
SPARC	miR‐1245b‐5p	Secreted protein acidic and cysteine rich
MMP15	miR‐1245b‐5p	Matrix metallopeptidase 15
PKP2	miR‐1245b‐5p	Plakophilin 2
ZEB2	miR‐1245b‐5p	Zinc finger E‐box binding homeobox 2
WNT4	miR‐1245b‐5p	Wnt family member 4
WNT6	miR‐1245b‐5p	Wnt family member 6
WNT7B	miR‐1245b‐5p	Wnt family member 7B
DLL1	miR‐1245b‐5p	Delta like canonical Notch ligand 1
JAG1	miR‐1245b‐5p	Jagged 1
CDKL2	miR‐1245b‐5p	Cyclin‐dependent kinase like 2
NOTCH1	miR‐1245b‐5p	Notch 1
TMPRSS4	miR‐1245b‐5p	Transmembrane serine protease 4
TUSC3	miR‐1245b‐5p	Tumor suppressor candidate 3
COL4A1	miR‐1245b‐5p	Collagen type IV alpha 1 chain
COL4A3	miR‐1245b‐5p	Collagen type IV alpha 3 chain
COL4A6	miR‐1245b‐5p	Collagen type IV alpha 6 chain
CLDN12	miR‐1245b‐5p	Claudin 12
CLDN7	miR‐1245b‐5p	Claudin 7
TGFB2	miR‐1245b, miR‐92a‐3p	Transforming growth factor beta 2
TGFBR2	miR‐1245b, miR‐92a‐3p	Transforming growth factor beta receptor 2
TRAF6	miR‐1245b, miR‐92a‐3p	TNF receptor associated factor 6
MAP2K6	miR‐1245b, miR‐92a‐3p	Mitogen‐activated protein kinase kinase 6
MAPK13	miR‐1245b, miR‐92a‐3p	Mitogen‐activated protein kinase 13
MAPK14	miR‐1245b, miR‐92a‐3p	Mitogen‐activated protein kinase 14
PIK3CA	miR‐1245b, miR‐92a‐3p	Phosphatidylinositol‐4,5‐bisphosphate 3‐kinase catalytic subunit alpha
AKT1	miR‐1245b, miR‐92a‐3p	AKT serine/threonine kinase 1
RBBP4	miR‐1245b, miR‐92a‐3p	RB binding protein 4, chromatin remodeling factor
WNT2B	miR‐1245b, miR‐92a‐3p	Wnt family member 2B
NOTCH2	miR‐1245b, miR‐92a‐3p	Notch 2
NOTCH4	miR‐1245b, miR‐92a‐3p	Notch 4

### 3.2 Expression analysis of miR‐1245b‐5p, miR‐92a‐3p, and *GATA3* in the breast samples

**FIGURE 7:**
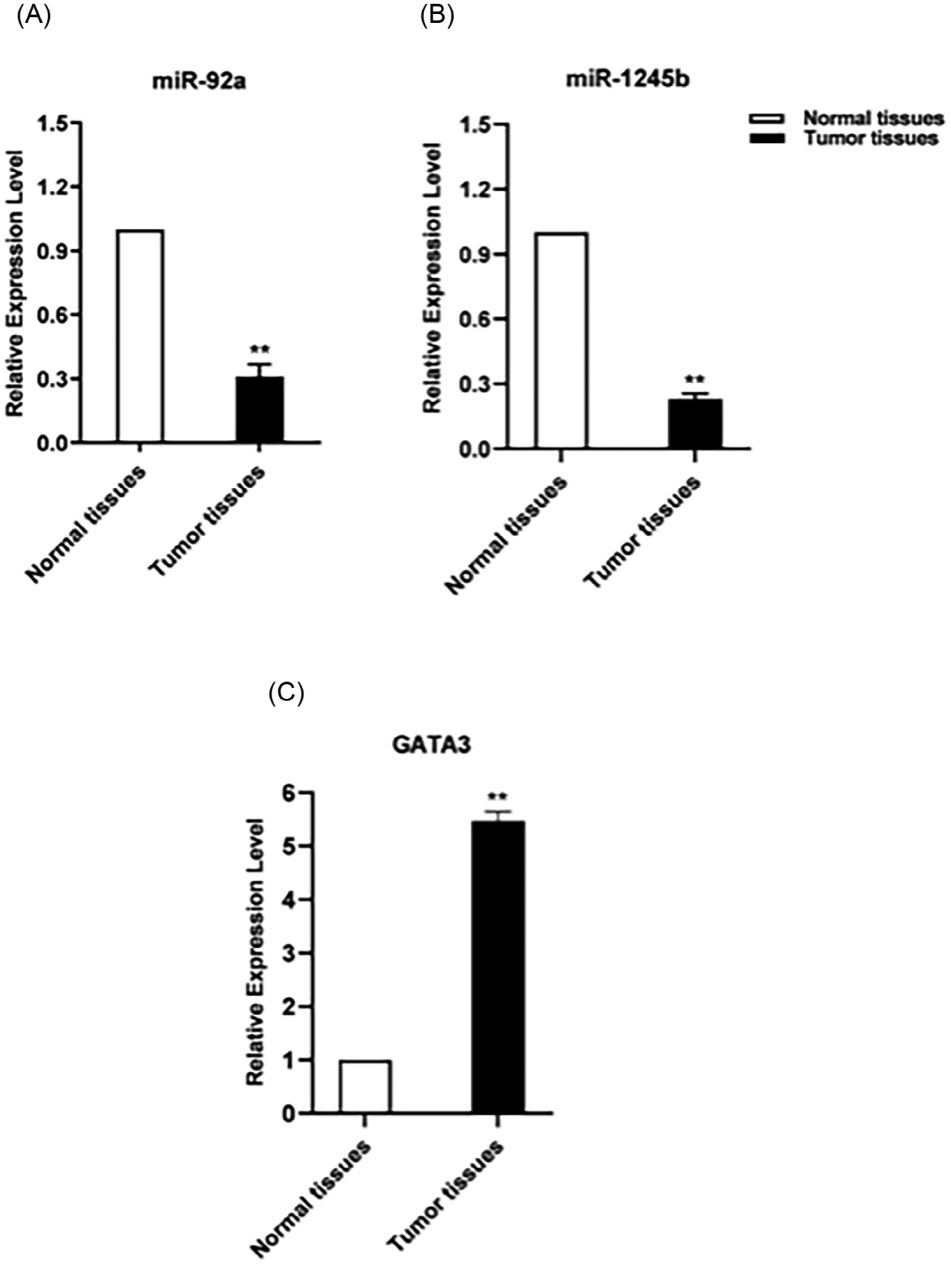
The expression levels of miR‐92a‐3p (A), miR‐1245b‐5p (B), and *GATA3* (C) are measured by the formula 2 ^(−ΔΔ CT)^, and fold changes are presented as mean ± standard deviation. The asterisk shows the statistical significance between patients and healthy controls (** *p* < 0.01).

Expression analysis of miR‐92‐3p and miR‐1245‐5p in the cancerous and surrounding normal breast tissue samples was performed by qRT‐PCR. A significant downregulation of miR‐92a‐3p in tumor tissues, especially in the primary grade compared to normal tissues was obtained (a− 3.22 fold reduction and *p* < .01) (Figure 7A). Moreover, the miR‐92a‐3p expression showed a significant correlation with differentiation grade, stage, and expression level of estrogen receptor (ER) in tumor tissue (*p* < .0001, *p* < .0001, and *p* < .001, respectively). The expression levels of miR‐1245‐5p were also significantly decreased in the cancer tissue compared to the surrounding normal tissues (−4.35‐fold, *p* < .01) (Figure 7B). Also, miR‐1245b‐5p showed a significant correlation with lymph node and ER status (*p* < .05) and not with stage/grade of patients with BC (*p* > .05). In addition, *GATA3* mRNA was upregulated 5.46 folds in BC tissues (*p* < .01) compared to healthy tissues (Figure 7C).

### 3.3 miRNAs and *GATA3* correlation with the clinical profile of BC

Analytical evaluation of the correlation of the *GATA3* expression with clinicopathological features revealed that the *GATA3* expression decreased (*p* < .001, *p* < .0001) as the grade and stage increase (namely tumor progression). Moreover, the expression level of *GATA3* was higher in ER^+^ compared to ER^−^ patients (*p* < .001), and in the Lamin A subgroup compared to the other subgroups (*p* < .0001). In regard to miR‐92‐3p, the expression of this miRNA further decreased in ER^−^ patients (*p* < .001). Furthermore, there was an inverse correlation between the grade, stage, and tumor size and the expression of this miRNA, suggesting that as the grade and stage increase, the miR‐92‐3p expression further decreased (*p* < .0001). Moreover, the expression of this miRNA in patients whose lymph nodes were involved was significantly reduced compared to patients whose lymph was not affected (*p* < .001). Among all the clinicopathological features, miR‐1245b‐5p was correlated with lymph and ER status (*p* < .05), and the expression of this miRNA decreased in ER negative patients and patients whose lymph was unaffected.

### 3.4 Correlation among miRNAs and *GATA3* expression

The relative expression levels of miR‐1245b‐5p and miR‐92a‐3p were compared to the *GATA3* expression in all samples using Pearson's correlation coefficient analysis. The results showed a slight correlation and statistical insignificance between the expression levels of miR‐92a‐3p and *GATA3* (Pearson's correlation = 0.3, *p* > .05). No correlation between miR‐1245b‐5p and *GATA3* was observed.

### 3.5 The ROC curve

**FIGURE 8:**
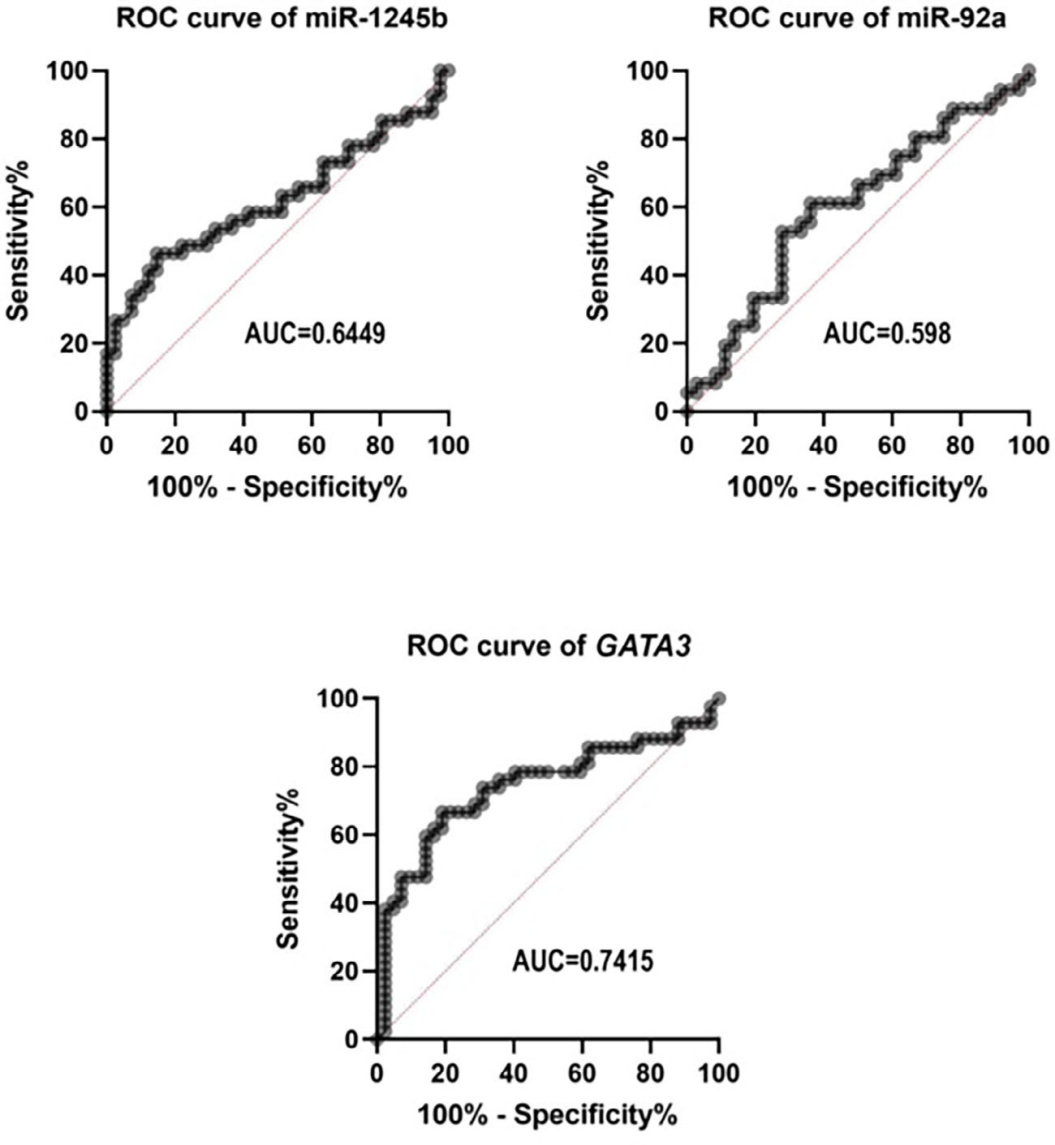
The discriminatory power of the individual miRNAs and *GATA3* for the detection of patients with BC and control individuals.

The ROC curve was conducted to determine the specificity and sensitivity of *GATA3* and the studied miRNAs during the differentiation of BC and control tissues. As shown in Figure 8, the area under the ROC curve (AUC) for *GATA3* corresponded to 0.7415 (*p* < .0001), with the specificity and sensitivity of 97.62% and 97.62%, respectively. The AUC of miR‐92a‐3p was calculated at 0.5980 (*p* = .1526), with the best specificity and sensitivity of 100% and 97.22%, respectively. The AUC of miR‐1245b‐5p was calculated at 0.6449 (*p* = .0239), with the best specificity and sensitivity of 97.56% and 97.56%, respectively.

## 4 DISCUSSION

BC is a serious global concern for the female population. Environmental, genetic, and epigenetic factors have been attributed to BC pathogenesis.^33^ Recent findings on the potential of miRNAs as biomarkers for various diseases among various populations, show that the miRNA expression varied among European‐Americans and African‐Americans populations with early‐stage BC.^34^ Orangi et al. found that miR‐34a and miR‐9 are predictive biomarkers for BC diagnosis in females.^35^ Furthermore, Nama et al. reported that miRNAs can be used as diagnostic and prognostic biomarkers in the Triple Negative BC stages (TNBC).^36^

Furthermore, miRNA‐based therapeutic studies have been carried out on various cancers. For example, miR‐497 was shown to target the HIF‐1a and VEGF, inhibit tumor growth and suppress angiogenesis in the BC.^37^ In addition, miR‐503 accompany with *GATA3*, targets *ZNF217* and suppresses prostate cancer.^38^ Via establishing the c‐SRC and Bcl2 signaling pathways, miR‐34a blocks tumor proliferation and invasion and thus induces senescence. It sensitizes mesenchymal‐TNBC cells to Dasatinib, thus inhibits tumor growth and suppresses cell migration.^39^ Guo et al. showed that the miR‐539 overexpression suppressed tumor growth in BC by targeting the EGF receptor.^40^ Liu et al. concluded that miR‐30e decreased metastasis, invasion, and chemotherapy resistance by suppressing IRSI.^8^ Because of the importance of miRNAs in the prediction, diagnosis, and treatment of various cancers,^9^, ^10^, ^41^, ^42^ we studied miR‐1245b‐5p and miR‐92a‐3p miRNAs in BC. Our bioinformatics results predicted that these two miRNAs could potentially bind to the 3’UTR region of *GATA3*. Furthermore, the expression profile of these miRNAs and their potential target gene (*GATA3*) was assessed using qRT‐PCR in the BC tissues. Moreover, the ROC curve analysis showed a significant diagnostic accuracy of miR‐1245b and *GATA3* between BC‐ patients and healthy individuals.

So far, the role of miR‐92a‐3p in BC has not been completely determined. The expression levels of this miRNA are even different in various breast tumor samples. Several studies have reported an increase or decrease in the expression of this miRNA. For example, a study showed that miR‐92a‐3p‐3p expression was higher in the tumor tissue than in the normal tissue and suggested that the increased expression of this miRNA was associated with poor prognosis of BC.^12^ In contrast, in our study, a decrease in the expression of this miRNA was observed in cancer tissues. Moreover, Moi et al. observed that the expression of miR‐92a was significantly associated with several clinicopathological features, such as the grade of BC specimens.^43^ As well as, using the in‐situ hybridization method, it was found that reducing the miR‐92a‐3p expression is inversely related to tumor grade and recurrence‐free survival (RFC).^44^ Another study showed that tumors with a low miR‐92a‐3p expression are associated with advanced tumor stages and poor patient survival.^23^ In agreement, our findings showed a significant invert correlation between the miR‐92a‐3p level and the grade, stage, and lymph node status. As, the decreased level of miR‐92a‐3p accompany with the increase of grade and stage of tumor and also involving lymph node in patients with BC. Furthermore, Cun et al. reported a significant association between tamoxifen resistance, and also ER expression with miR‐92a‐3p expression in BC cells.^12^ In agreement, our results showed a decreased expression of miR‐92a‐3p in ER^−^ BC patients. However, we not found a significant correlation between the miR‐92a level and tumor size.

The activity and contribution of miR‐1245b‐5p in BC pathogenesis, as well as EMT and invasion, has yet to be fully characterized. Yang et al. found that the upregulation of miR‐1245 was associated with breast and lung cancer progression.^45^ Another study found that upregulation of the c‐Myc induced the miR‐1245 expression, leading to BRACA2 suppression in BC.^25^ Furthermore, the upregulation of miR‐1245 has been shown to reduce NKG2D receptor expression in natural killer cells.^46^ Also, miR‐1245 has been shown to accelerate colon cancer cell invasion and proliferation by targeting BRACA2.^47^ Weiyan Lou et al. demonstrated that miR‐1245b‐5p was downregulated in drug resistance BC patients.^48^ In agreement, our data showed the decreased expression of miR‐1245b‐5p in BC tissues compared to surrounding health tissues. Moreover, the assessment of clinical factors showed that the miR‐1245b‐5p expression was reversely associated with lymph vascular invasion and ER expression in BC patients. However, no significant correlation was found between other clinical factors and the miR‐1245b‐5p expression.

*GATA3* is a transcription factor with function in the morphogenesis, differentiation, and proliferation of luminal epithelial cells in breast tissues.^49^, ^50^, ^51^, ^52^ The *GATA3* expresses in epithelial cells but not specifically in ductal cells.^53^, ^54^, ^55^ Deregulation of the *GATA3* has been reported in various cancers.^18^, ^56^, ^57^, ^58^, ^59^ A high level of *GATA3* expression has been reported in luminal types of BC.^60^ Mehra et al. showed the expression of *GATA3* was significantly related to the stage, grade, lymph node, tumor size, and the ER expression. Moreover, the highest level of *GATA3* expression was reported in the luminal A subtype of BC samples.^54^, ^61^, ^62^ In agreement, our results showed that the expression of *GATA3* in the luminal subtype of BC was higher than that in other subtypes. However, the *GATA* expression was higher in the BC‐affected patients with lymph node involvement than in BC‐affected patients without lymph node involvement.

In addition, using in silico tools, several target genes of miR‐92a‐3p and miR‐1245b involved in EMT and invasion pathways were predicted. Our results confirmed the potential contribution of studied miRNAs in regulating these signaling pathways. Moreover, the gene ontology analysis by DIANA miRPath v. 3 tools showed several pathways related to metabolism, biosynthesis, and transcription that potentially targeted by these miRNAs. These in silico results support the possible involvement of these miRNAs in BC pathogenesis. Despite the limitations of this study including the low fund to evaluate a greater number of miRNAs and target genes related to BC, it is hoped that this study can be effective to provide novel information in future on the predictive diagnostic and therapeutic efficacy of these biomarkers and target genes in various types of cancer.

## 5 CONCLUSION

Our findings showed an increased level of *GATA3* expression, as well as, a decreased level of miR‐1245b‐5p and miR‐92a‐3p expression in BC tissues. Moreover, bioinformatics analysis confirmed miR‐1245b‐5p and miR‐92a‐3p as potential regulators of signaling pathways involved in BC pathogenesis especially invasion and EMT pathways. So, it seems that these genetic markers can be effective in the diagnosis and treatment of BC and other types of cancer.

## AUTHOR CONTRIBUTIONS

**Mahtab Yadollahi Farsani:** Data curation (lead); formal analysis (lead); investigation (lead); validation (lead); writing – original draft (lead). **Zeinab Amini Farsani:** Methodology (lead); project administration (lead); validation (lead). **Shohreh Teimuri:** Visualization (lead); writing – review and editing (equal). **Mohsen Kolahdouzan:** Resources (equal). **Reza Eshraghi Samani:** Resources (equal). **Hossein Teimori:** Conceptualization; project administration; supervision.

## FUNDING INFORMATION

This work was supported by Shahrekord University of Medical Sciences (Grant# 3847).

## CONFLICT OF INTEREST STATEMENT

The authors declare that they have no conflicts of interest.

## ETHICS STATEMENT

The Research Ethics Committee of Shahrekord University of Medical Sciences has approved the plan (IR.SKUMS.REC.1397‐267).

## Data Availability

Data available on request from the authors.
